# Molecular mechanism of β-arrestin-2 pre-activation by phosphatidylinositol 4,5-bisphosphate

**DOI:** 10.1038/s44319-024-00239-x

**Published:** 2024-09-06

**Authors:** Kiae Kim, Ka Young Chung

**Affiliations:** School of Pharmacy, Sungkyunkwan University, 2066 Seobu-ro, Jangan-gu, Suwon, 16419 Republic of Korea

**Keywords:** Arrestin, Phosphatidylinositol 4,5-bisphosphate, Structure, HDX-MS, Membranes & Trafficking, Signal Transduction, Structural Biology

## Abstract

Phosphorylated residues of G protein-coupled receptors bind to the N-domain of arrestin, resulting in the release of its C-terminus. This induces further allosteric conformational changes, such as polar core disruption, alteration of interdomain loops, and domain rotation, which transform arrestins into the receptor-activated state. It is widely accepted that arrestin activation occurs by conformational changes propagated from the N- to the C-domain. However, recent studies have revealed that binding of phosphatidylinositol 4,5-bisphosphate (PIP_2_) to the C-domain transforms arrestins into a pre-active state. Here, we aimed to elucidate the mechanisms underlying PIP_2_-induced arrestin pre-activation. We compare the conformational changes of β-arrestin-2 upon binding of PIP_2_ or phosphorylated C-tail peptide of vasopressin receptor type 2 using hydrogen/deuterium exchange mass spectrometry (HDX-MS). Introducing point mutations on the potential routes of the allosteric conformational changes and analyzing these mutant constructs with HDX-MS reveals that PIP_2_-binding at the C-domain affects the back loop, which destabilizes the gate loop and βXX to transform β-arrestin-2 into the pre-active state.

## Synopsis

The mechanism of β-arrestin (βarr2) pre-activation by PIP_2_ has not yet been fully understood. This study suggests that PIP_2_ induces allosteric conformational changes in βarr2, which ultimately affect the conformational dynamics of the βXX strand.

- PIP_2_ increases the conformational dynamics of the βXX strand, indicating the pre-activation of βarr2.
- HDX-MS and mutational studies have revealed the allosteric structural pathway from the PIP_2_-binding sites to the βXX strand.
- Conformational perturbation of the back loop through the gate loop of βarr2 is the allosteric structural pathway of the PIP_2_-induced pre-activation of βarr2.

## Abstract (web summary)

The mechanism of β-arrestin (βarr2) pre-activation by PIP_2_ has not yet been fully understood. This study suggests that PIP_2_ induces allosteric conformational changes in βarr2, which ultimately affect the conformational dynamics of the βXX strand.

## Graphical abstract

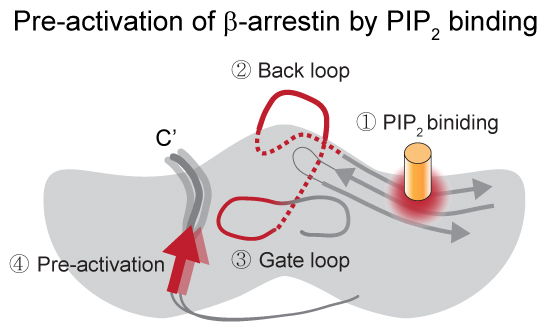

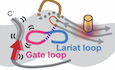

## Introduction

Arrestins, a protein family regulating G protein-coupled receptor (GPCR) signaling, have four distinct members in mammals (arrestin-1–4) (Benovic et al, 1987; Lohse et al, 1990). Arrestin-1 and -4 are visual system-specific, while arrestin-2 and -3 (β-arrestin-1 [βarr1] and 2 [βarr2]) are widely expressed (Lohse and Hoffmann, 2014). They desensitize and internalize agonist-activated phosphorylated GPCRs (Benovic et al, 1987) and regulate other signaling pathways (Coffa et al, 2011; Park et al, 2019; Perry-Hauser et al, 2022; Perry et al, 2019; Qu et al, 2021a; Smith and Rajagopal, 2016; Srivastava et al, 2015). Understanding how arrestins are activated at the structural and molecular level is crucial for the development of drugs targeting GPCRs or related pathways.

**Figure 1:**
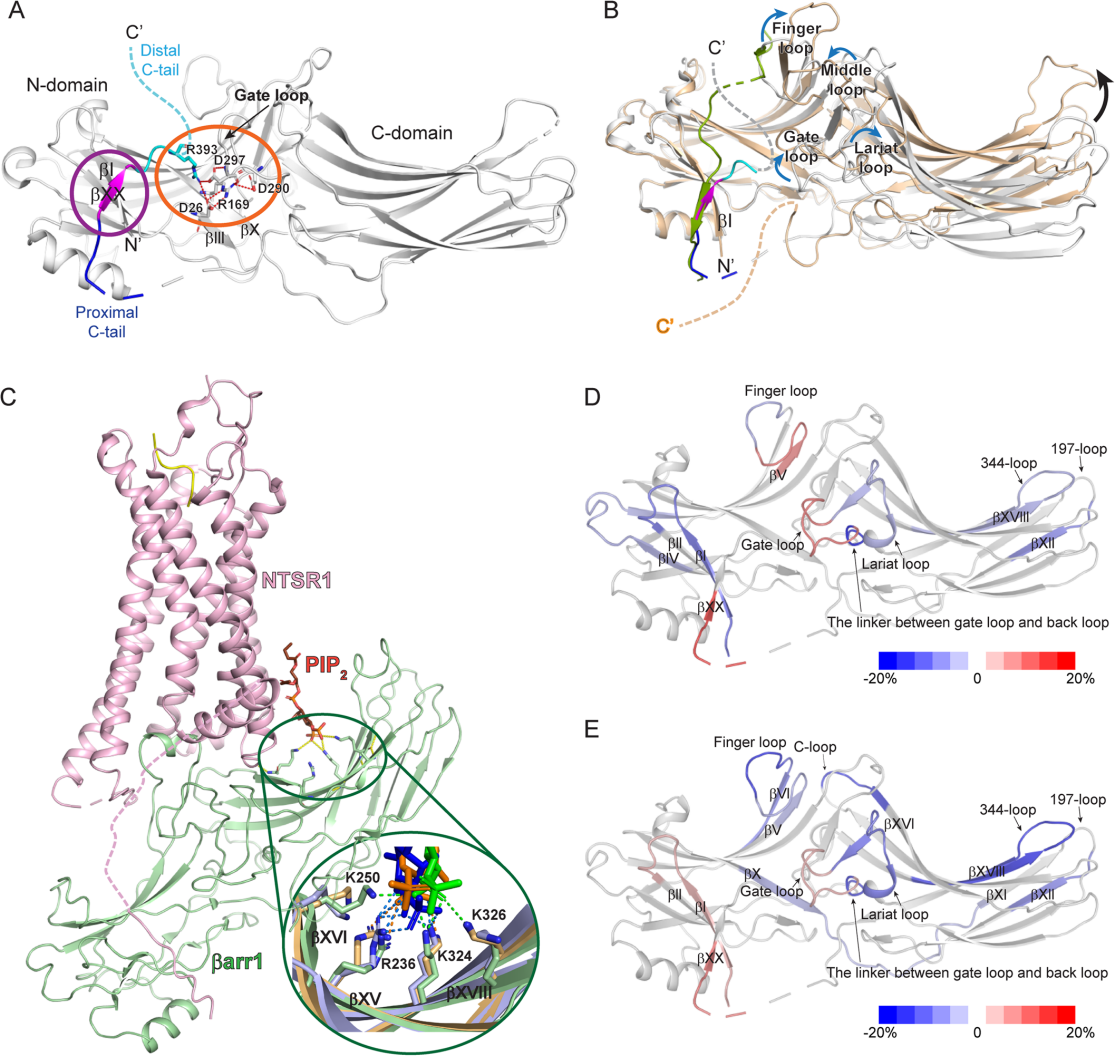
Structures of βarr in various states and HDX-MS profile changes upon the binding of V2Rpp or PIP_2_ to βarr2. (**A**) Structure of βarr1 in the basal state (PDB: 1G4R) (Data ref: Han et al, 2001a; Han et al, 2001b). The basal state βarr1 is colored gray with the C-terminus colored blue (proximal C-tail), magenta (βXX), and cyan (distal C-tail). Unresolved regions are indicated by dotted lines. The interaction between βXX and the residues in the N-domain is indicated in the purple circle, and the polar core is denoted in the orange circle. Residues that are involved in the polar core formation are shown as sticks. (**B**) Comparison of the structure of βarr1 in basal (PDB: 1G4R) (Data ref: Han et al, 2001a; Han et al, 2001b) and V2Rpp-bound (PDB: 4JQI) (Data ref: Shukla et al, 2013a; Shukla et al, 2013b) states. V2Rpp-bound βarr1 is colored light orange and V2Rpp is colored green. The color codes for the basal state of βarr1 are same as those of (**A**). The conformational changes of the loop regions are shown with blue arrows, and the domain rotation is indicated with a black arrow. (**C**) Structure of the NTSR1-βarr1 complex (PDB: 6UP7) (Data ref: Huang et al, 2020a; Huang et al, 2020b). NTSR1 is colored light pink, and βarr1 is colored light green. PIP_2_ is indicated with orange sticks. The residues that interact with PIP_2_ are shown as sticks. In the enlarged green circle, various modes of interaction between βarr1 and PIP_2_ are shown; PIP_2_ in the NTSR1-βarr1 complex (PDB: 6UP7) (Data ref: Huang et al, 2020a; Huang et al, 2020b) is colored orange, the interacting residues in βarr1 are colored light orange, and the ionic interactions between PIP_2_ and βarr1 are shown as green dotted lines; PIP_2_ in the GCGR1-βarr1 complexes (PDB: 8JRU and 8JRV) (Data ref: Chen et al, 2023a; Chen et al, 2023b) is colored blue or green, the interacting residues in βarr1 are colored light blue or light green, and the ionic interactions between PIP_2_ and βarr1 are shown as blue or orange dotted lines respectively. (**D**) HDX-MS profile comparison between the apo and V2Rpp-bound βarr2. The HDX-level differences (i.e., HDX levels of apo βarr2–HDX levels of V2Rpp-bound βarr2) are color-coded on the basal state structure of βarr2 (PDB: 3P2D) (Data ref: Zhan et al, 2011a; Zhan et al, 2011b). Results were derived from three independent experiments. (**E**) HDX-MS profile comparison between the basal and PIP_2_-bound βarr2. The HDX-level differences (i.e., HDX levels of apo βarr2–HDX levels of PIP_2_-bound βarr2) are color-coded on the basal state structure of βarr2 (PDB: 3P2D) (Data ref: Zhan et al, 2011a; Zhan et al, 2011b). Results were derived from three independent experiments (biological). The color-coded HDX-level differences are based on the maximum differences at any D_2_O incubation time point. The detailed HDX-MS data are summarized in Dataset EV1 and Fig. EV2.

Previous studies revealed arrestin structures in basal and receptor-bound active states (Chen et al, 2023b; Hirsch et al, 1999; Huang et al, 2020b; Lee et al, 2020; Mayer et al, 2019; Park et al, 2019; Shukla et al, 2014; Staus et al, 2020; Yang et al, 2015; Yun et al, 2015; Zhou et al, 2017). Arrestins consist of N- and C-domains with a seven-stranded β sandwich in each domain (Fig. 1A). The basal state is stabilized by the interaction between the C-tail, more precisely βXX, and N-domain (Fig. 1A, purple circle) and the polar core formed by ionic interactions between residues within the gate loop, βIII, βX, and C-tail (Fig. 1A, orange circle).

**Figure EV2:**

Deuterium uptake plots of selective peptides of WT βarr2 with or without V2Rpp or PIP_2_ co-incubation. Results were derived from three independent experiments. The statistical significance of the differences was determined using Student’s t test (*P < 0.05). Exact *p*-values are provided in Dataset EV1. Data are presented as mean ± standard error of the mean. Black or grey * indicates statistically significant difference between apo WT βarr2 and PIP_2_-bound WT βarr2 or V2Rpp-bound WT βarr2, respectively. Smaller symbols indicate each data point.

Binding of the phosphorylated GPCR (Fig. 1B, green) at the N-domain transforms arrestins into the active state by releasing βXX, disrupting the polar core, and affecting the conformation of the loops between the N- and C-domains, and altering the relative interdomain orientation (Fig. 1B). Although these conformational changes are the “canonical” changes of the receptor-activated arrestins, the degree of these changes can vary depending on the receptor types and phosphorylation patterns, resulting in different arrestin active states and functional outcomes (Kaya et al, 2020; Latorraca et al, 2020; Maharana et al, 2023b; Mayer et al, 2019; Yang et al, 2015; Zhou et al, 2017).

In recent years, plasma membrane components including phosphatidylinositol 4,5-bisphosphate (PIP_2_) have been implicated in βarr activation (Eichel et al, 2018; Grimes et al, 2023; Huang et al, 2020b; Janetzko et al, 2022; Kang et al, 2015; Zhai et al, 2023). High-resolution structures of GPCR-βarr complexes show βarr’s C-domain contacting lipids or detergents (Chen et al, 2023b; Staus et al, 2020), which facilitates GPCR-βarr complex formation (Lally et al, 2017; Zhou et al, 2017). The involvement of PIP_2_ in βarr activation has been extensively suggested. With the assistance of PIP_2_, βarr can become “catalytically activated” (i.e., active without receptor binding) (Eichel et al, 2018). A subsequent study proposed that PIP_2_-binding is necessary for certain GPCR-βarr interactions and that PIP_2_ promotes βarr activation (Janetzko et al, 2022). Notably, the cryo-electron microscopy structures showed PIP_2_-binding at the C-domain of βarr1 in the neurotensin receptor 1 (NTSR1)-βarr1 and glucagon receptor (GCGR)-βarr1 complexes (Fig. 1C) (Chen et al, 2023b; Huang et al, 2020b). However, the precise structural mechanism by which PIP_2_ promotes arrestin activation remains unclear.

Nevertheless, only few studies examined PIP_2_-induced βarr conformational changes by labeling specific residues with a fluorophore or ^19^F (Janetzko et al, 2022; Zhai et al, 2023). Here, we investigated PIP_2_-induced arrestin activation mechanism using hydrogen-deuterium exchange mass spectrometry (HDX-MS). HDX-MS monitors the exchange between the amide hydrogen in the protein and deuterium in the solvent, providing information about the protein conformational dynamics (Bai et al, 1993; Mayne, 2016). We compared the conformational dynamics of the PIP_2_-induced and the phosphorylated C-tail peptide of the vasopressin receptor type 2 (V2Rpp)-induced active states of βarr2 and explored activation mechanisms through mutational studies.

## Results and discussion

### Conformational changes of βarr2 upon PIP_2_-binding

To investigate PIP_2_-induced conformational changes of βarr2, purified βarr2 was incubated with water-soluble PIP_2_ (150 μM) as described in the Methods. Subsequently, deuterium exchange was initiated on ice for various durations (10, 100, 1000, and 10,000 s). The peptic peptides used for the HDX-MS analyses are shown in Fig. EV1, and the HDX-MS data analyzed in the present study are summarized in Dataset EV1. To compare the PIP_2_-induced structural changes with phosphorylated GPCR-induced changes, we also examined the effects of V2Rpp (500 μM), a well-established model system for understanding βarr interactions with phosphorylated receptor C-tails (Fig. 1B) (Latorraca et al, 2020; Mayer et al, 2019; Shukla et al, 2013b; Yang et al, 2015).

**Figure EV1:**

Sequence coverage map of wild-type β-arrestin-2 (βarr2). The blue bars indicate analyzed peptic peptides.

V2Rpp induced higher HDX in the N-terminal half of the finger loop, gate loop, and proximal C-tail through βXX (Figs. 1D and EV2, peptides 62–69, 292–302, and 382–389), and lower HDX in a few regions within the N-domain (βI, βIV through βV, and C-terminal half of the finger loop; peptides 1–19, 41–55, and 70–76), domain interfaces (the lariat loop and the linker between the gate loop and back loop; peptides 281–291 and 303–306), and a few regions within the C-domain (197-loop and βXVIII; peptides 195–201 and 324–338) (Figs. 1D and EV2).

These HDX-MS data well-reflected the known V2Rpp-induced conformational changes of βarr2. V2Rpp (Fig. 1B, green) interacts at the N-domain groove and near βI. Thus, lower HDX levels of the V2Rpp-bound βarr2 at the N-domain (specifically, βI and C-terminal half of the finger loop) probe the V2Rpp-binding in these regions. In addition, V2Rpp-induced higher HDX levels in the gate loop and proximal C-tail through βXX indicate conformational changes resulting from βXX release and polar core disruption. The HDX-level changes at the domain interfaces suggest conformational changes in the loop regions at the domain interfaces and/or domain rotation upon V2Rpp-binding, and the changes at the C-domain may reflect long-range allosteric conformational changes transmitted from the N-domain V2Rpp-binding site.

As HDX-MS analysis effectively probed the V2Rpp-induced activation of βarr2, we sought to analyze the PIP_2_-induced conformational changes of βarr2. Based on the NTSR1-βarr1 and GCGR-βarr1 complex structures, PIP_2_ can interact with positively charged residues at βXV (R236 in the βarr1 sequence), βXVI (K250 in the βarr1 sequence), and βXVIII (K324 and K326 in the βarr1 sequence) (Fig. 1C, inlet). The HDX-MS analysis revealed that the HDX levels at βXVIII become lower upon co-incubation with PIP_2_ (Figs. 1E and EV2, peptide 324–338), implying the binding of PIP_2_ to βarr2. However, the HDX levels of the peptides covering βXV (Figs. 1E and EV2, peptide 219–239) and βXVI (Figs. 1E and EV2, peptide 251–258) were not affected. This may be due to three reasons. First, as HDX monitors the buffer exposure of the amide hydrogens at the peptide backbone, HDX levels could not be affected if the binding occurs through the charge–charge interaction mediated by the amino acid side chains without altering the peptide backbone conformation. Second, the PIP_2_-interacting residues may differ slightly between the receptor-bound (i.e., the NTSR1-βarr1 and GCGR-βarr1 complexes) and unbound states (i.e., current study). Even in the receptor-bound states, PIP_2_ interacted differently between the NTSR1-bound and GCGR-bound states (Fig. 1C, inlet). Third, the reported βarr structures with PIP_2_ are βarr1 structures (Fig. 1C), but in this study, we analyzed the conformation of βarr2. Therefore, the differences may stem from variations between these subtypes. Nevertheless, the HDX-MS data indicate that PIP_2_ interacts at the positively charged region within the C-domain of βarr2.

Interestingly, PIP_2_ induced higher HDX levels at βI, gate loop, and proximal C-tail through βXX (Figs. 1E and EV2, peptides 1–19, 292–302, and 382–389), which is the canonical feature of the βarr activation (i.e., βXX release and polar core disruption) (Fig. 1B) (Kim et al, 2015; Shukla et al, 2014; Yun et al, 2015). Of note, the HDX levels of the PIP_2_-bound state at the gate loop and proximal C-tail through βXX were still lower than the V2Rpp-bound state (Fig. EV2, peptides 292–302 and 382–389), which suggests that the PIP_2_-bound state is not as fully active as the V2Rpp-bound state. Thus, these results suggest that the binding of PIP_2_ destabilizes the gate loop and the interaction of βXX at the N-domain, which may transform βarr2 more amenable to be activated (i.e., pre-active state).

PIP_2_-induced HDX-level changes were also evident at the finger loop, βVI, βX through βXI, 197-loop, C-loop, lariat loop, and the linker between the gate loop and back loop (Figs. 1E and EV2, peptides 62–69, 70–76, 75–81, 168–186, 195–201, 246–250, 281–291, and 303–306). Although most of these regions were also affected by V2Rpp-binding, the HDX-MS profiles at the finger loop and its extension (i.e., βVI) (Figs. 1E and EV2, peptides 62–69, 70–76, and 75–81) and the lariat and gate loops (Figs. 1E and EV2, peptides 281–291 and 292–302) differed between the V2Rpp- and PIP_2_-bound states, suggesting that these regions adopt different conformations between V2Rpp- and PIP_2_-bound states. Furthermore, βX through βXI and the C-loop were affected by PIP_2_, but not by V2Rpp (Figs. 1E and EV2, peptides 168–186 and 246–250).

### Distal C-tail of βarr2 is not involved in PIP_2_-induced pre-activation

The HDX-MS data suggest that the interaction of PIP_2_ at the C-domain affects the conformational dynamics of βI, gate loop, and βXX (Fig. 1E) potentially through the allosteric transmission of the conformational changes from the C-domain to the gate loop and βXX. Thus, we sought to understand the routes for the allosteric conformational changes transmitted from the PIP_2_-binding sites to the gate loop or βXX.

**Figure 2:**
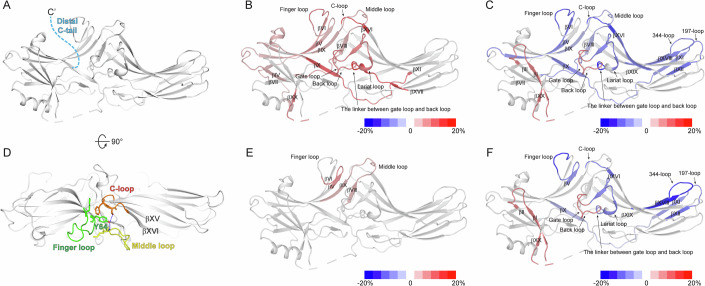
HDX-MS profile analysis of β-arrestin-2 (βarr2)_1-394 and Y64. (**A**) The truncated distal C-tail of βarr2 is colored light blue on the basal state structure of βarr2 (PDB: 3P2D) (Data ref:Zhan et al, 2011a; Zhan et al, 2011b). (**B**) HDX-MS profile comparison between the WT and βarr2_1-394. The HDX-level differences (i.e., HDX levels of WT βarr2–HDX levels of βarr2_1-394) are color-coded on the basal state structure of βarr2 (PDB: 3P2D) (Data ref:Zhan et al, 2011a; Zhan et al, 2011b). Results were derived from three independent experiments (biological). (**C**) HDX-MS profile comparison between apo and PIP_2_-bound βarr2_1-394. The HDX-level differences (i.e., HDX levels of apo βarr2_1-394 – HDX levels of PIP_2_-bound βarr2_1-394) are color-coded on the basal state structure of βarr2 (PDB: 3P2D) (Data ref:Zhan et al, 2011a; Zhan et al, 2011b). Results were derived from three independent experiments (biological). (**D**) The top-view of the interaction between the finger, middle, and C-loops of βarr2 in the basal state (PDB: 3P2D) (Data ref:Zhan et al, 2011a; Zhan et al, 2011b). Y64 is indicated by green sticks. The finger, middle, and C-loops are green, yellow, and orange, respectively. (**E**) HDX-MS profiles of the WT and Y64A. HDX-level differences (i.e., HDX levels of WT βarr2–HDX levels of Y64A) are color-coded based on the basal state structure of βarr2 (PDB: 3P2D) (Data ref:Zhan et al, 2011a; Zhan et al, 2011b). Results were derived from three independent experiments (biological). (**F**) HDX-MS profile comparison of apo- and PIP_2_-bound Y64A. The HDX-level differences (i.e., HDX levels of apo Y64A–HDX levels of PIP_2_-bound Y64A) are color-coded based on the basal state structure of βarr2 (PDB: 3P2D) (Data ref:Zhan et al, 2011a; Zhan et al, 2011b). Results were derived from three independent experiments (biological). The color-coded HDX-level differences are based on the maximum differences at any D_2_O incubation time point. The detailed HDX-MS data are summarized in Dataset EV1 and Figs. EV3 and EV4.

The initial candidate was the distal C-tail (Fig. 2A). High-resolution structures have not fully characterized the distal C-tail because it is often unresolved or truncated (Han et al, 2001b; Hirsch et al, 1999; Zhan et al, 2011b). Nonetheless, given that the truncation of the distal C-tail transforms βarrs into the pre-active state (Celver et al, 2002; Gurevich, 1998; Gurevich et al, 1997; Kovoor et al, 1999), it is reasonable to hypothesize that the binding of PIP_2_ perturbs the conformational dynamics of the distal C-tail to impact the activation status of βarrs. To test this hypothesis, we truncated the distal C-tail (βarr2_1-394) and examined PIP_2_-induced HDX-level changes. If the distal C-tail serves as the route for allosteric conformational changes, PIP_2_ should not affect HDX levels at the gate loop or βXX in βarr2_1-394.

**Figure EV3:**
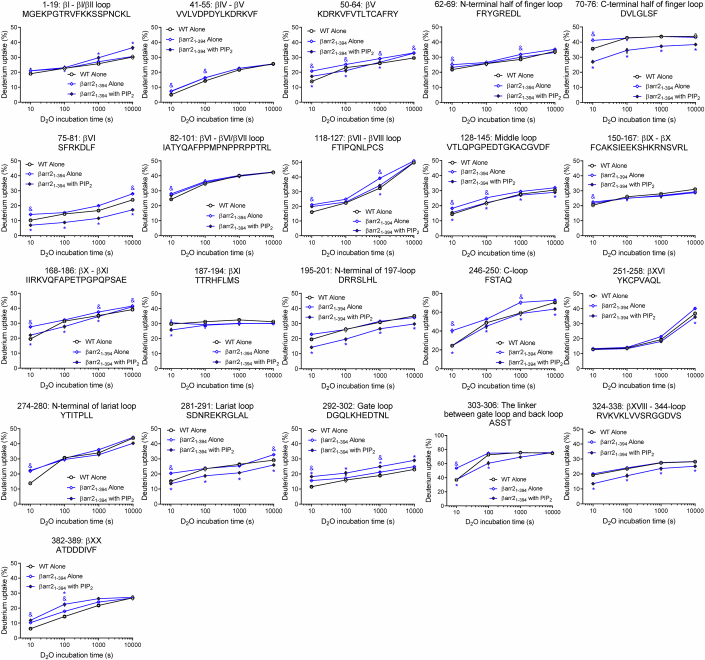
Deuterium uptake plots of selective peptides of apo WT βarr2 and βarr2_1-394 with or without PIP_2_ co-incubation. Results were derived from three independent experiments. The statistical significance of the differences was determined using Student’s *t* test (^&,^*P < 0.05). Exact *p*-values are provided in Dataset EV1. & indicates statistically significant difference between apo WT βarr2 and apo βarr2_1-394. * indicates statistically significant difference between apo and PIP_2_-bound βarr2_1-394. Data are presented as mean ± standard error of the mean. Smaller symbols indicate each data point.

**Figure EV4:**
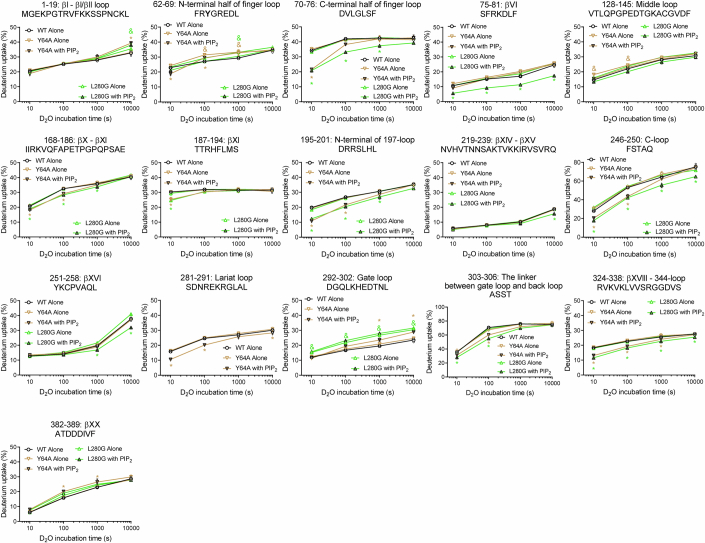
Deuterium uptake plots of selective peptides of apo WT βarr2 and Y64A and L280G with or without PIP_2_ co-incubation. Results were derived from three independent experiments. The statistical significance of the differences was determined using Student’s *t* test (^&,^*P < 0.05). Exact *p*-values are provided in Dataset EV1. Yellow green & indicates statistically significant difference between apo WT βarr2 and apo Y64A or L280G, respectively. Yellow or green * indicates statistically significant difference between apo and PIP_2_-bound Y64A or L280G, respectively. Data are presented as mean ± standard error of the mean. Smaller symbols indicate each data point.

In the apo state, compared to the wild-type (WT), βarr2_1-394 exhibited higher HDX levels in numerous regions across the N- and C-domains (Figs. 2B and EV3), indicating that the distal C-tail truncation yields βarr2 conformationally more dynamic. This increased conformational dynamics, especially at the gate loop and βXX, accounts for the pre-active state, as previously reported (Celver et al, 2002; Gurevich, 1998; Gurevich et al, 1997; Kovoor et al, 1999).

PIP_2_ induced HDX-level changes of βarr2_1-394 in the regions similar to the WT (compare Figs. 1E and 2C; Table EV1). Decreased HDX levels were detected at the PIP_2_-binding site (Figs. 2C and EV3, peptide 324–338) and increased HDX levels were detected at βI, gate loop, and proximal C-tail through βXX (Fig. 2C and EV3, peptides 1–19, 292–302, and 382–389). These findings suggest that the PIP_2_ can induce further activation of βarr2_1-394.

Other regions altered in the WT were also similarly affected (Figs. 2C and EV3, peptides 70–76, 75–81, 168–186, 195–201, 246–250, 281–291, and 303–306). A few other regions where we did not observe HDX changes with PIP_2_-bound WT were also affected (Fig. EV3, peptides 50–64, 118–127, 128–145, and 251–258), but the HDX levels of these regions became statistically no different to those of the WT (Fig. EV3, peptides 128–145 and 251–258) or similar to those of the WT (Fig. EV3, peptides 50–64 and 118–127). In addition, we observed the decreased HDX levels at βXI (Figs. 2C and EV3, peptide 187–194). Overall, the HDX profile changes of the PIP_2_-bound βarr2_1-394 (Fig. 2C) were similar to those of the PIP_2_-bound WT (Fig. 1E), suggesting that the distal C-tail is not the route for allosteric conformational changes from the PIP_2_-binding sites to the gate loop or βXX.

### Y64 in the finger loop is not involved in PIP_2_-induced pre-activation

The finger, middle, and C-loops between the N- and C-domains undergo dramatic conformational changes upon activation (Fig. 1B) and interact with the cytosolic core of the receptor (Fig. 1C) (Huang et al, 2020b; Kang et al, 2015). In the basal state, the finger, middle, and C-loops form a designated structure through hydrophobic and polar interactions (Fig. 2D). PIP_2_ altered HDX levels in the finger loop and C-loop (Fig. 1E). Notably, the C-loop is located at the C-domain as an extension from the PIP_2_-binding sites (βXV and βXVI) (Figs. 1C and 2D). Therefore, our second hypothesis was that interactions between the finger, middle, and C-loops transmit the allosteric conformational changes. In the basal state, Y64 is located in a pocket formed by the finger-, middle-, and C-loops (Fig. 2D), probably stabilizing the interactions between these three loops. Thus, we reasoned that the mutation of Y64 destabilizes the interactions between these three loops and breaks off the transmission route from the PIP_2_-binding sites.

In the apo state, the mutation of Y64 to alanine (Y64A) altered HDX levels in the N-terminal half of the finger loop and middle loop compared to those in the WT (Figs. 2E and EV4, peptides 62–69 and 128–145), reflecting a disturbance of the conformation surrounding Y64, as expected. Upon addition of PIP_2_, Y64A displayed HDX changes in the regions similar to those of the WT (compare Figs. 1E and 2F; Table EV1). HDX levels were decreased at the PIP_2_-binding site (Figs. 2F and EV4, peptide 324–338) and increased at βI, gate loop, and proximal C-tail through βXX (Figs. 2F and EV4, peptides 1–19, 292–302, and 382–389). These findings suggest that PIP_2_ can induce pre-activation of Y64A. Other regions altered in the WT were also affected (Figs. 2F and EV4, peptides 62–69, 70–76, 168–186, 195–201, 246–250, 281–291, and 303–306). In addition, we observed decreased HDX levels at βXI (Fig. 2F and EV4, peptides 187– 194). These results suggest that the interactions between the finger, middle, and C-loops are not routes for allosteric conformational transmission.

### The lariat loop of βarr2 is involved in PIP_2_-induced pre-activation

**Figure 3:**
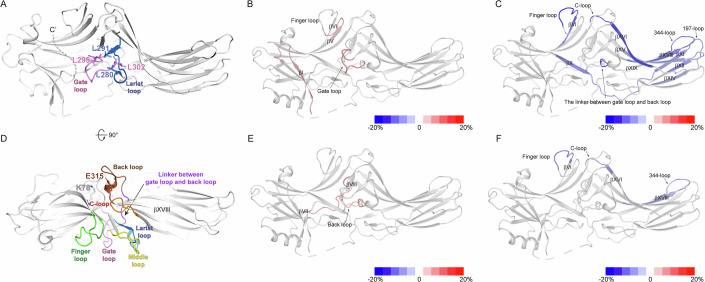
HDX-MS profile analysis of L280 and E315. (**A**) Interaction between the lariat and gate loops in the basal state βarr2 (PDB: 3P2D) (Data ref:Zhan et al, 2011a; Zhan et al, 2011b). The lariat and gate loops are shown in blue and pink, respectively. The hydrophobic residues forming the interaction between the lariat and gate loops are shown as sticks. (**B**) HDX-MS profile comparison of the WT and L280G. The HDX-level differences (i.e., HDX levels of WT βarr2–HDX levels of L280G) are color-coded on the basal state structure of βarr2 (PDB: 3P2D) (Data ref:Zhan et al, 2011a; Zhan et al, 2011b). Results were derived from three independent experiments (biological). (**C**) HDX-MS profile comparison of apo and PIP_2_-bound L280G. The HDX-level differences (i.e., HDX levels of apo Y64A–HDX levels of PIP_2_-bound L280G) are color-coded on the basal state structure of βarr2 (PDB: 3P2D) (Data ref:Zhan et al, 2011a; Zhan et al, 2011b). Results were derived from three independent experiments (biological). (**D**) Top-view of the structure of the basal state βarr2 (PDB: 3P2D) (Data ref:Zhan et al, 2011a; Zhan et al, 2011b) showing relative positions of the back (brown), gate (pink), and lariat (blue) loops, and the linker between the gate and back loops (violet). E315 is shown as brown sticks, and K78 is shown as gray sticks. The finger, middle, and C-loops are colored green, yellow, and orange, respectively. (**E**) HDX-MS profile comparison between the WT and E315A. The HDX-level differences (i.e., HDX levels of WT βarr2–HDX levels of E315A) are color-coded on the basal state structure of βarr2 (PDB: 3P2D) (Data ref:Zhan et al, 2011a; Zhan et al, 2011b). Results were derived from three independent experiments (biological). (**F**) HDX-MS profile comparison of apo and PIP_2_-bound E315A. The HDX-level differences (i.e., HDX levels of apo Y64A–HDX levels of PIP_2_-bound E315A) are color-coded on the basal state structure of βarr2 (PDB: 3P2D) (Data ref:Zhan et al, 2011a; Zhan et al, 2011b). Results were derived from three independent experiments (biological). The color-coded HDX-level differences are based on the maximum differences at any D_2_O incubation time point. The detailed HDX-MS data are summarized in Dataset EV1 and Figs. EV4 and EV5.

Because the distal C-tail and the interactions between the finger, middle, and C-loops do not serve as routes for allosteric conformational transmission, we sought other potential routes. After careful examination of the basal state structure and HDX-MS data of PIP_2_-bound βarr2 (Figs. 1E and EV2), L280 in the lariat loop and E315 in the back loop were chosen as potential key residues (Fig. 3).

**Figure EV5:**
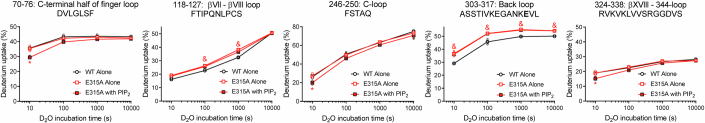
Deuterium uptake plots of selective peptides of apo WT βarr2 and E315A with or without PIP_2_ co-incubation. Results were derived from three independent experiments. The statistical significance of the differences was determined using Student’s *t* test (^&,^*P < 0.05). Exact *p*-values are provided in Dataset EV1. & indicates statistically significant difference between apo WT βarr2 and apo E315A. * indicates statistically significant difference between apo and PIP_2_-bound E315A. Data are presented as mean ± standard error of the mean. Smaller symbols indicate each data point.

In the basal state, L280 faces the gate loop and forms hydrophobic interactions with L291, L295, and L302 (Fig. 3A), which stabilizes the conformation of the gate and lariat loops. If the allosteric conformational transmission is mediated through perturbation of the interaction of the lariat and gate loops, the L280 mutation would disrupt this route. To test this hypothesis, we mutated L280 to glycine.

In the apo state, L280G showed altered HDX levels at the gate loop (Figs. 3B and EV4, peptide 292–302), reflecting the altered conformation near the lariat and gate loop regions due to the mutation. L280G mutation also altered HDX levels at βI and the N-terminal half of the finger loop (Figs. 3B and EV4, peptides 1–19 and 62–69). The results suggest that perturbation of the interaction between the gate and the lariat loops could alter the conformational dynamics of remote regions, such as βI and finger loop.

Upon PIP_2_-binding to L280G, we observed decreased HDX levels at the PIP_2_-binding interface (Figs. 3C and EV4, peptide 324–338). We also observed altered HDX at the regions similar to those of the WT (compare Figs. 1E and 3C; Table EV1), such as the C-terminal half of the finger loop, βVI, βX through βXI, 197-loop, C-loop, and the linker between lariat loop and back loop (Figs. 3C and EV4, peptides 70–76, 75–81, 168–186, 195–201, 246–250, and 303–306). In addition, decreased HDX levels were evident at βXI, βXIV through βXV, and βXVI (Figs. 3C and EV4, peptides 187–194, 219–239, and 251–258).

In contrast, changes in HDX levels for βI, the gate loop, and proximal C-tail through βXX were not evident upon the binding of PIP_2_ to L280G (Figs. 3C and EV4, peptides 1–19, 292–302, and 382–389). Therefore, the binding of PIP_2_ in L280G induces conformational changes in most regions similar to those of the WT but failed to transform it to the pre-active conformation (i.e., disturbance of the gate loop and βXX), suggesting that perturbation of the interaction of the lariat and gate loops is the route for the transmission of the conformational changes from the PIP_2_-binding site to βXX.

### The back loop of βarr2 is involved in PIP_2_-induced pre-activation

Another potential route we examined was the back loop. Although the HDX-MS profiles of the back loop were not affected by PIP_2_, the neighboring C-loop and the linker between the lariat and back loops were altered (Figs. 1E and EV2, peptides 246–250 and 303–306). Interestingly, the back loop is an extension of the PIP_2_-binding sites (βXVIII), located adjacent to the C-loop, and directly connected to the gate loop through the linker between the gate loop and the back loop (Fig. 3D). Previous evidence suggested that in the basal state, E315 at the back loop occasionally forms salt bridge with K78 at βVI (Fig. 3D) and that disruption of this interaction results in ligand-independent accumulation of βarr2 in the clathrin-coated endocytic structures (Eichel et al, 2018). Moreover, the back loop has been reported as a potential route for the conformational transition from PIP_2_-binding to βarr1 C-tail release (Zhai et al, 2023). Therefore, we further examined the role of the back loop in the PIP_2_-induce βarr2 activation.

To test this hypothesis, we mutated E315 to alanine, which would break the interaction between E315 and Y78 (Fig. 3D). In the apo state, compared to the WT, E315A showed higher HDX levels at the back loop and its neighboring βVII/βVIII loop (Figs. 3E and EV5, peptides 118–127 and 303–317) reflecting altered conformational dynamics of the back loop upon E315A mutation. As we did not observe HDX differences in other regions remote from the back loop, the results imply that the disruption of the interaction between E315 and K78 alters the local conformational dynamics but does not affect the overall conformational dynamics of βarr2.

Although the apo state did not show HDX-level differences between the WT and E315A other than in the back loop and βVII/βVIII loop, the effects of PIP_2_ on E315A were dramatically different from those on the WT (compare Figs. 1E and 3F; Table EV1). Although PIP_2_ induced a decrease in HDX levels at the PIP_2_-binding site in E315A (Figs. 3F and EV5, peptide 324–338), indicating PIP_2_-binding to E315A, we observed HDX-MS profile changes only within very limited regions, such as the C-terminal half of the finger loop and the C-loop (Figs. 3F and EV5, peptides 70–76 and 246–250) but no other regions. These results suggest that, in E315A, PIP_2_ could alter the C-loop and its neighboring finger loop but fails to transform βarr2 to the pre-active state. Thus, we conclude that the PIP_2_-induced conformational changes are allosterically transmitted through the back loop to βXX.

### PIP_2_ facilitates V2Rpp-induced βarr2 activation

**Figure 4:**
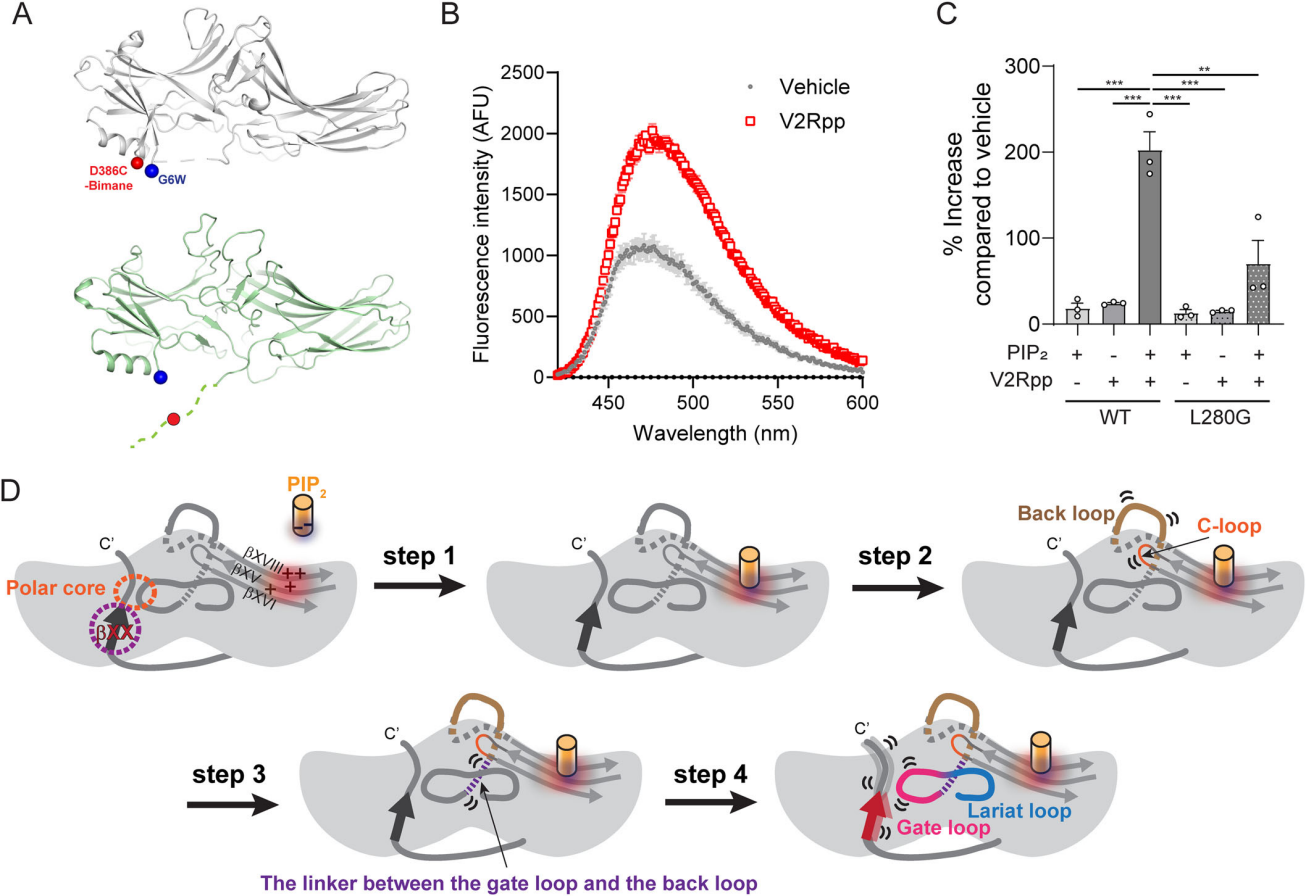
Proposed molecular mechanism of βarr2 pre-activation upon PIP_2_-binding. (**A**) The scheme of the experimental system analyzing C-tail release. The position of residues 6 and 386 are shown as red and blue spheres in the basal state structure of βarr2 (upper panel, PDB: 3P2D) (Data ref:Zhan et al, 2011a; Zhan et al, 2011b) and V2Rpp-bound structure of βarr2 (lower panel, PDB: 8GOC) (Data ref:Maharana et al, 2023a; Maharana et al, 2023b). (**B**) Bimane fluorescence traces of basal and V2Rpp-bound states. (**C**) Bimane fluorescence changes of wild-type (WT) or L280G βarr2 upon addition of PIP_2_, V2Rpp, or PIP_2_ pre-incubation followed by V2Rpp addition. The statistical significance of the differences was determined using one-way ANOVA followed by Tukey’s posttest (**P < 0.001 and ***P < 0.0001). Exact *P*-values between PIP_2_-WT vs. PIP_2_-V2Rpp-WT, V2Rpp-WT vs. PIP_2_-V2Rpp-WT, PIP_2_-V2Rpp-WT vs. PIP_2_-L280G, PIP_2_-V2Rpp-WT vs. V2Rpp-L280G, and PIP_2_-V2Rpp-WT vs. PIP_2_-V2Rpp-L280G are 0.000017, 0.000016, 0.000009, 0.000010, and 0.000317, respectively. Results were derived from three independent experiments (biological). Data are presented as mean ± standard error of the mean. (**D**) βArr2 is shown schematically as a gray shape, particular regions are highlighted in dark gray (dotted) lines. Secondary structures (beta sheets) of interest are shown as arrows. The positively charged region in C-domain is indicated by red color and “+“ symbols. PIP_2_ is shown as the orange cylinder. In its basal state, βarr2 is stabilized through the interaction of βXX at the N-domain (dotted purple circle) and through the ionic interactions between N- and C-domains (i.e., polar core, dotted orange circle). The negatively charged PIP_2_ binds to the positively charged region within the C-domain of βarr2 (step 1). This PIP_2_-binding induces alterations in the conformational dynamics of the back and C-loops (step 2). The resulting conformational change is transmitted to the linker between the gate loop and the back loop (step 3). Consequently, the conformational dynamics of the gate and lariat loops are altered, leading to the destabilization of the gate loop and βXX, ultimately resulting in the pre-activation of βarr2 (step 4).

A recent study by Zhai et al reported that the simultaneous binding of V2Rpp and PIP_2_ induces complex conformational changes in different structural regions (Zhai et al, 2023). Here, we tested whether pre-incubation with PIP_2_ affects the V2Rpp-induced C-tail release. To examine the C-tail release of βarr2, we developed an experimental system using bimane fluorophore, an environment-sensitive fluorescent molecule. We substituted glycine at residue 6 to tryptophan (G6W) and labeled bimane at the βarr2 C-tail by substituting aspartate at residue 386 with cysteine (D386C) (Fig. 4A) in the cysteine-free βarr2 background (Cys-free βarr2: C17S/C60A/C126S/C141I/C151V/C244V/C253V/C271S/C405S/C410S). Bimane fluorescence can be quenched by nearby tryptophan residues (Jones Brunette and Farrens, 2014). Therefore, in the basal state, bimane fluorescence at the residue 386 is quenched by the tryptophan at the residue 6 (Fig. 4A, upper panel), but upon C-tail release, quenching is abolished as the residue 386 moves away from the residue 6 (Fig. 4A, lower panel).

When we incubated the bimane-labeled βarr2 with excess V2Rpp (300 μM), bimane fluorescence increased (Fig. 4B), reflecting the C-tail release. To examine the pre-activation effect of PIP_2_, we reduced V2Rpp concentration to 30 μM, where it induces minimal bimane fluorescence increase (Fig. 4C). Similarly, 30 μM PIP_2_ did not induce C-tail release (Fig. 4C). However, pre-incubation with PIP_2_ followed by V2Rpp addition significantly increased the bimane fluorescence (Fig. 4C), implying that PIP_2_ pre-incubation facilitates V2Rpp-induced βarr2 activation.

Introducing the L280G mutation to disrupt the allosteric conformational pathway reduced the augmentation of the V2Rpp-induced C-tail release after PIP_2_ pre-incubation (Fig. 4C). This result confirms that the lariat loop is the allosteric conformational change route through which PIP_2_ facilitates phosphorylated receptor-induced βarr2 activation.

### Proposed mechanism of the PIP_2_-induced βarr2 activation

Here, we comprehensively analyzed the conformational dynamics of the whole βarr2 using HDX-MS. Our data confirmed that PIP_2_-bound βarr2 adopts the pre-active conformation, enhancing βarr2’s interaction with V2Rpp. Interestingly, the PIP_2_-binding sites are remote from βXX and polar core (Fig. 4D), suggesting allosteric conformational transmission. The HDX-MS data suggested that both L280G and E315A failed to activate βarr2, but the two mutants exhibited different PIP_2_-induced conformational changes (Fig. 3). In L280G, PIP_2_ could still induce conformational changes in almost all the regions similar to the WT, except the gate loop, βXX, and βI (Fig. 3C). In contrast, in E315A, the binding of PIP_2_ induced conformational changes in only limited regions (i.e., the C-loop and its neighboring finger loop; Fig. 3F) without affecting other regions. Thus, we propose that the back loop precede the gate loop when the allosteric conformational changes are transmitted from the PIP_2_-binding site to βXX. In summary, the binding of PIP_2_ at the C-domain (Fig. 4D, step 1) affects the loops (i.e., the back loop and C-loop) that are connected to the PIP_2_-binding β-strands (Fig. 4D, step 2). The altered conformational dynamics of the back loop is allosterically transmitted to the lariat and gate loops to pre-activate βarr2 (Fig. 4D, step 4) through the linker between the gate and back loops (Fig. 4D, step 3).

### Limitations and future perspectives

This study highlights the structural mechanism of the PIP_2_-induced βarr2 pre-activation but has limitations. First, within the cell, arrestins interact with a variety of other components, including phospholipids, receptors, G proteins, and signaling proteins (Chen et al, 2023b; Grimes et al, 2023; Lally et al, 2017; Qu et al, 2021b; Smith et al, 2021). Therefore, the PIP_2_- or V2Rpp-induced conformational changes of the purified βarr2 might be too simplistic compared to the complex nature within the cell. Second, our study couldn’t detail the allosteric conformation changes at the atomic level. Advances in biophysical techniques, such as time-resolved Cryo-EM (Klebl et al, 2023), could provide deeper insights into the step-by-step conformational changes at the atomic level.

It has long been believed that the interaction of the phosphorylated GPCRs at the N-domain is the key process for arrestin activation (Edward Zhou et al, 2019; Gusach et al, 2023; Hilger et al, 2018; Maharana et al, 2022; Seyedabadi et al, 2021; Wisler et al, 2014; Zhao et al, 2017). However, now it is emerging that arrestin activation can be achieved through various processes. Inositol hexaphosphate (IP_6_) interacts at the phosphate sensor within the N-domain resulting βXX release to activate βarr2 and triggers further downstream signal transduction (Chen et al, 2017). PIP_2_ has been suggested to interact at the C-domain to activate arrestins (Janetzko et al, 2022; Zhai et al, 2023), and here we further propose the structural mechanism of PIP_2_-induced arrestin pre-activation. As it is evident that arrestins can be activated via various routes, it is needed to investigate the structural differences and the functional consequences of the different active states of arrestins.

## Methods

**Table:** Reagents and tools table

Reagent/resource	Reference or source	Identifier or catalog number
**Experimental models**		
*E. coli* DH5α Chemically Competent	Enzynomics	Cat# CP010
*E. coli* ROSETTA(DE3)	Novagen	Cat# 70954
**Recombinant DNA**		
pET28a-WT rat β-arrestin-2	This study	N/A
pET28a-β-arrestin 2_1-394	This study	N/A
pET28a-β-arrestin 2_Y64A	This study	N/A
pET28a-β-arrestin 2_L280G	This study	N/A
pET28a-β-arrestin 2_E315A	This study	N/A
pET28b-rat β-arrestin-2 _Cysfree_G6W_D386C	This study	N/A
pET28b-rat β-arrestin-2 _Cysfree_G6W_L280G_D386C	This study	N/A
**Oligonucleotides and other sequence-based reagents**		
PCR primer: Y64A Forward: GTGCCTTCCGCGCTGGCCGAGAAGACCTGGATG	Bionics	N/A
PCR primer: L280G Forward: CACCATAACCCCGCTGGGCAGTGACAACCGAGAGAAG	Bionics	N/A
PCR primer: E315A Forward: GAGGGAGCCAACAAGGCGGTGCTGGGAATCCTAG	Bionics	N/A
PCR primer: G6W Forward: CATATGGGTGAGAAGCCCTGGACCAGGGTCTTCAAG	Bionics	N/A
PCR primer: D386C Forward: CCAACTATGCCACAGACGACTGCATCGTGTTTGAGGAC	Bionics	N/A
**Chemicals, enzymes and other reagents**		
Protease inhibitor cocktail	BioVision	Cat# K272
Deuterium oxide	Cambridge isotope laboratories	Cat# DLM-11-100
Ni-IDA resin	Cytiva	Cat# 17057501
Dimethyl sulfoxide	Duchefa	Cat# D1370
Leupeptin	Goldbio	Cat# L-010
Lysozyme	Goldbio	Cat# L-040
TCEP	Goldbio	Cat# TCEP
IPTG	Goldbio	Cat# I2481C
Kanamycin	Goldbio	Cat# K-120-10N
Chloramphenicol	Duchefa	Cat# C0113.0025
Pepsin column	Life Technologies	Cat# 2313100
DNase I	Roche	Cat# 11284932001
Benzamidine	Sigma-Aldrich	Cat# 12072
Imidazole	Sigma-Aldrich	Cat# I2399
08:0 PI(4,5)P_2_	Avanti Polar Lipids	Cat# 850185
Zeba Desalt Spin Desalting Columns	Thermo Scientific	Cat# 89890
Bromobimane	MedChemExpress	Cat# HY-100041
V2Rpp	Tufts University Core Facility	N/A
**Software**		
Prism 8.0	Graphpad	graphpad.com
PyMol 2.3	Schrodinger	pymol.org
Proteinlynx Global Server 2.4	Waters	www.waters.com
DynamX 3.0	Waters	www.waters.com

### βarr2 expression and purification

All protein constructs for HDX-MS were cloned into the pET28a vector, and mutant rat βarr2 constructs for Trp-induced bimane fluorescence quenching experiments were cloned into the pET28b. The rat βarr2 constructs were transformed into *Escherichia coli* BL21 (DE3). Point mutations were prepared using site-directed mutagenesis. Expression and purification were performed as previously described (Park et al, 2019). Briefly, WT rat βarr2 and the mutants were grown in LB broth medium at 37 °C until the optical density at 600 nm reached 0.4–0.6. The bacteria were then induced with 30 μM IPTG for 24 h at 16 °C. Proteins were purified using Ni-IDA resins and size-exclusion chromatography.

### Hydrogen/deuterium exchange

βarr2 at a final concentration of 50 μM in 20 mM HEPES pH 7.4, 150 mM NaCl, and 100 μM Tris [2-carboxyethyl] phosphine hydrochloride was co-incubated with 500 μM V2Rpp or 150 μM PIP_2_ for 1 h at room temperature. HDX was performed by mixing 2 μL of protein (50 μM) with 28 μL of D_2_O buffer (20 mM HEPES pH 7.4, 150 mM NaCl, 100 μM Tris [2-carboxyethyl] phosphine hydrochloride, and 10% glycerol in D_2_O) and incubating the mixture for 10, 100, 1000, and 10,000 s on ice. At the indicated time points, the reaction was quenched by adding 30 μL of ice-cold quench buffer (60 mM NaH_2_PO_4_ pH 2.01 and 10% glycerol) and snap-frozen on dry ice. Identical procedures were conducted for nondeuterated samples using a H_2_O buffer comprising 20 mM HEPES, pH 7.4, 150 mM NaCl, and 10% glycerol in H_2_O.

### HDX-MS

HDX-MS and data analyses were conducted as previously described (Du et al, 2019; Qu et al, 2021a). Briefly, the quenched samples underwent online digestion by passage through an immobilized pepsin column (2.1 × 30 mm; Life Technologies, Carlsbad, CA, USA). The digested peptide fragments were collected on a C18 VanGuard trap column (1.7 mm × 30 mm; Waters, Milford, MA, USA), followed by ultra-pressure liquid chromatography using an ACQUITY UPLC C18 column (1.7 mm, 1.0 mm × 100 mm; Waters). All settings and conditions for the system, such as voltage, temperature, collision energy, and lockspray, were as previously reported (Du et al, 2019; Qu et al, 2021a). Peptic peptides from nondeuterated samples were identified using ProteinLynx Global Server 2.4 (Waters). To process HDX-MS data, the amount of deuterium in each peptide was determined by measuring the centroid of the isotopic distribution using DynamX 3.0 (Waters).

### Trp-induced bimane fluorescence quenching experiment

βarr2 was prepared at a final concentration of 8 μM or 20 μM in 20 mM HEPES pH 7.4, 150 mM NaCl, and co-incubated with a 10-fold molar excess of bromobimane for 1 h on ice. Excess dye was removed by buffer exchange using a desalting column. Then, βarr2 at a final concentration of 3 μM was co-incubated with V2Rpp for 1 h at room temperature, with or without pre-incubation of PIP_2_. The samples were placed in a MicroFluor 96-well fluorescent plate. The samples were excited at 390 nm, and the emitted fluorescence was measured from 420 to 600 nm using 1-nm step size by Synergy Neo or Synergy Neo2 (BioTek, Winooski, VT, USA).

### Statistical analysis

For HDX-MS analysis, mass differences >0.22 Da and 2% were considered significant. Student’s *t* test was used to determine the statistically significant differences between individual time points. For Trp-induced bimane fluorescence quenching data, the significant differences were analyzed by one-way ANOVA followed by Tukey’s posttest. The statistical analyses were performed by GraphPad Prism software (GraphPad, San Diego, CA, USA), and statistical significance was set at *P* < 0.05.

## Supplementary information

Table EV1

Peer Review File

Dataset EV1

Expanded View Figures

## Data Availability

HDX-MS data have been deposited to ProteomeXchange Consortium via PRIDE43 partner repository with the set identifier PXD049391 (https://www.ebi.ac.uk/pride/archive/projects/PXD049391). The source data of this paper are collected in the following database record: biostudies:S-SCDT-10_1038-S44319-024-00239-x.

## References

[CR2] Bai Y, Milne JS, Mayne L, Englander SW (1993) Primary structure effects on peptide group hydrogen exchange. Proteins: Struct Funct Bioinforma 17:75–86 10.1002/prot.340170110 PMC3438223 8234246

[CR3] Benovic JL, Kühn H, Weyand I, Codina J, Caron MG, Lefkowitz RJ (1987) Functional desensitization of the isolated beta-adrenergic receptor by the beta-adrenergic receptor kinase: potential role of an analog of the retinal protein arrestin (48-kDa protein). Proc Natl Acad Sci USA 84:8879–8882 2827157 10.1073/pnas.84.24.8879 PMC299654

[CR4] Celver J, Vishnivetskiy SA, Chavkin C, Gurevich VV (2002) Conservation of the phosphate-sensitive elements in the arrestin family of proteins. J Biol Chem 277:9043–9048 11782458 10.1074/jbc.M107400200

[CR5] Chen K, Zhang C, Lin S, Yan X, Cai H, Yi C, Ma L, Chu X, Liu Y, Zhu Y et al (2023a) Protein Data Bank. Structure of the glucagon receptor bound to beta-arrestin 1 in ligand-free state. https://www.rcsb.org/structure/8JRU

[CR6] Chen K, Zhang C, Lin S, Yan X, Cai H, Yi C, Ma L, Chu X, Liu Y, Zhu Y et al (2023b) Tail engagement of arrestin at the glucagon receptor. Nature 620:904–910 37558880 10.1038/s41586-023-06420-x PMC10447241

[CR7] Chen Q, Perry NA, Vishnivetskiy SA, Berndt S, Gilbert NC, Zhuo Y, Singh PK, Tholen J, Ohi MD, Gurevich EV et al (2017) Structural basis of arrestin-3 activation and signaling. Nat Commun 8:1427 29127291 10.1038/s41467-017-01218-8 PMC5681653

[CR8] Coffa S, Breitman M, Hanson SM, Callaway K, Kook S, Dalby KN, Gurevich VV (2011) The effect of arrestin conformation on the recruitment of c-Raf1, MEK1, and ERK1/2 activation. PLoS ONE 6:e28723 22174878 10.1371/journal.pone.0028723 PMC3236217

[CR9] Du Y, Duc NM, Rasmussen SGF, Hilger D, Kubiak X, Wang L, Bohon J, Kim HR, Wegrecki M, Asuru A et al (2019) Assembly of a GPCR-G protein complex. Cell 177:1232–1242.e1211 31080064 10.1016/j.cell.2019.04.022 PMC6763313

[CR10] Edward Zhou X, Melcher K, Eric Xu H (2019) Structural biology of G protein-coupled receptor signaling complexes. Protein Sci 28:487–501 30311978 10.1002/pro.3526 PMC6371222

[CR11] Eichel K, Jullié D, Barsi-Rhyne B, Latorraca NR, Masureel M, Sibarita J-B, Dror RO, von Zastrow M (2018) Catalytic activation of β-arrestin by GPCRs. Nature 557:381–386 29720660 10.1038/s41586-018-0079-1 PMC6058965

[CR12] Grimes J, Koszegi Z, Lanoiselee Y, Miljus T, O’Brien SL, Stepniewski TM, Medel-Lacruz B, Baidya M, Makarova M, Mistry R et al (2023) Plasma membrane preassociation drives beta-arrestin coupling to receptors and activation. Cell 186:2238–2255 e2220 37146613 10.1016/j.cell.2023.04.018 PMC7614532

[CR13] Gurevich VV (1998) The selectivity of visual arrestin for light-activated phosphorhodopsin is controlled by multiple nonredundant mechanisms. J Biol Chem 273:15501–15506 9624137 10.1074/jbc.273.25.15501

[CR14] Gurevich VV, Pals-Rylaarsdam R, Benovic JL, Hosey MM, Onorato JJ (1997) Agonist-receptor-arrestin, an alternative ternary complex with high agonist affinity. J Biol Chem 272:28849–28852 9360951 10.1074/jbc.272.46.28849

[CR15] Gusach A, García-Nafría J, Tate CG (2023) New insights into GPCR coupling and dimerisation from cryo-EM structures. Curr Opin Struct Biol 80:102574 36963163 10.1016/j.sbi.2023.102574 PMC10423944

[CR16] Han M, Gurevich VV, Vishnivetskiy SA, Sigler PB, Schubert C (2001a) Protein Data Bank. Structure of bovine beta-arrestin 1 (https://www.rcsb.org/structure/1G4R) [DATASET] 10.1016/s0969-2126(01)00644-x 11566136

[CR17] Han M, Gurevich VV, Vishnivetskiy SA, Sigler PB, Schubert C (2001b) Crystal structure of β-arrestin at 1.9 Å: possible mechanism of receptor binding and membrane translocation. Structure 9:869–880 11566136 10.1016/s0969-2126(01)00644-x

[CR18] Hilger D, Masureel M, Kobilka BK (2018) Structure and dynamics of GPCR signaling complexes. Nat Struct Mol Biol 25:4–12 29323277 10.1038/s41594-017-0011-7 PMC6535338

[CR19] Hirsch JA, Schubert C, Gurevich VV, Sigler PB (1999) The 2.8 A crystal structure of visual arrestin: a model for arrestin’s regulation. Cell 97:257–269 10219246 10.1016/s0092-8674(00)80735-7

[CR20] Huang W, Masureel M, Qu Q, Janetzko J, Inoue A, Kato HE, Robertson MJ, Nguyen KC, Glenn JS, Skiniotis G et al (2020a) Protein Data Bank. Structure of neurotensin receptor and beta-arrestin 1 complex. (https://www.rcsb.org/structure/6UP7) [DATASET] 10.1038/s41586-020-1953-1 PMC7100716 31945771

[CR21] Huang W, Masureel M, Qu Q, Janetzko J, Inoue A, Kato HE, Robertson MJ, Nguyen KC, Glenn JS, Skiniotis G et al (2020b) Structure of the neurotensin receptor 1 in complex with β-arrestin 1. Nature 579:303–308 31945771 10.1038/s41586-020-1953-1 PMC7100716

[CR22] Janetzko J, Kise R, Barsi-Rhyne B, Siepe DH, Heydenreich FM, Kawakami K, Masureel M, Maeda S, Garcia KC, von Zastrow M et al (2022) Membrane phosphoinositides regulate GPCR-β-arrestin complex assembly and dynamics. Cell 185:4560–4573.e4519 36368322 10.1016/j.cell.2022.10.018 PMC10030194

[CR23] Jones Brunette AM, Farrens DL (2014) Distance mapping in proteins using fluorescence spectroscopy: tyrosine, like tryptophan, quenches bimane fluorescence in a distance-dependent manner. Biochemistry 53:6290–6301 25144569 10.1021/bi500493r PMC4196733

[CR24] Kang Y, Zhou XE, Gao X, He Y, Liu W, Ishchenko A, Barty A, White TA, Yefanov O, Han GW et al (2015) Crystal structure of rhodopsin bound to arrestin by femtosecond X-ray laser. Nature 523:561–567 26200343 10.1038/nature14656 PMC4521999

[CR25] Kaya AI, Perry NA, Gurevich VV, Iverson TM (2020) Phosphorylation barcode-dependent signal bias of the dopamine D1 receptor. Proc Natl Acad Sci USA 117:14139–14149 32503917 10.1073/pnas.1918736117 PMC7321966

[CR26] Kim DK, Yun Y, Kim HR, Seo M-D, Chung KY (2015) Different conformational dynamics of various active states of β-arrestin1 analyzed by hydrogen/deuterium exchange mass spectrometry. J Struct Biol 190:250–259 25871523 10.1016/j.jsb.2015.04.006

[CR27] Klebl DP, Aspinall L, Muench SP (2023) Time resolved applications for Cryo-EM; approaches, challenges and future directions. Curr Opin Struct Biol 83:102696 37716094 10.1016/j.sbi.2023.102696

[CR28] Kovoor A, Celver J, Abdryashitov RI, Chavkin C, Gurevich VV (1999) Targeted construction of phosphorylation-independent β-arrestin mutants with constitutive activity in cells. J Biol Chem 274:6831–6834 10066734 10.1074/jbc.274.11.6831

[CR29] Lally CC, Bauer B, Selent J, Sommer ME (2017) C-edge loops of arrestin function as a membrane anchor. Nat Commun 8:14258 28220785 10.1038/ncomms14258 PMC5321764

[CR30] Latorraca NR, Masureel M, Hollingsworth SA, Heydenreich FM, Suomivuori C-M, Brinton C, Townshend RJL, Bouvier M, Kobilka BK, Dror RO (2020) How GPCR phosphorylation patterns orchestrate arrestin-mediated signaling. Cell 183:1813–1825.e1818 33296703 10.1016/j.cell.2020.11.014 PMC7901245

[CR31] Lee Y, Warne T, Nehmé R, Pandey S, Dwivedi-Agnihotri H, Chaturvedi M, Edwards PC, García-Nafría J, Leslie AGW, Shukla AK et al (2020) Molecular basis of β-arrestin coupling to formoterol-bound β1-adrenoceptor. Nature 583:862–866 32555462 10.1038/s41586-020-2419-1 PMC7115876

[CR32] Lohse MJ, Benovic JL, Codina J, Caron MG, Lefkowitz RJ (1990) β-Arrestin: a protein that regulates β-adrenergic receptor function. Science 248:1547–1550 2163110 10.1126/science.2163110

[CR33] Lohse MJ, Hoffmann C (2014) Arrestin interactions with G protein-coupled receptors. In: Gurevich VV (ed) Arrestins—pharmacology and therapeutic potential. Springer Berlin Heidelberg, Berlin, Heidelberg, pp 15–56 10.1007/978-3-642-41199-1_2 24292823

[CR34] Maharana J, Banerjee R, Yadav MK, Sarma P, Shukla AK (2022) Emerging structural insights into GPCR–β-arrestin interaction and functional outcomes. Curr Opin Struct Biol 75:102406 35738165 10.1016/j.sbi.2022.102406 PMC7614528

[CR35] Maharana J, Sarma P, Yadav MK, Saha S, Singh V, Saha S, Chami M, Banerjee R, Shukla AK (2023a) Protein Data Bank. Structure of beta-arrestin2 in complex with a phosphopeptide corresponding to the human Vasopressin V2 receptor, V2R. (https://www.rcsb.org/structure/8GOC) [DATASET]

[CR36] Maharana J, Sarma P, Yadav MK, Saha S, Singh V, Saha S, Chami M, Banerjee R, Shukla AK (2023b) Structural snapshots uncover a key phosphorylation motif in GPCRs driving β-arrestin activation. Mol Cell 83:2091–2107.e2097 37209686 10.1016/j.molcel.2023.04.025 PMC7615930

[CR37] Mayer D, Damberger FF, Samarasimhareddy M, Feldmueller M, Vuckovic Z, Flock T, Bauer B, Mutt E, Zosel F, Allain FHT et al (2019) Distinct G protein-coupled receptor phosphorylation motifs modulate arrestin affinity and activation and global conformation. Nat Commun 10:1261 30890705 10.1038/s41467-019-09204-y PMC6424980

[CR38] Mayne L (2016) Chapter thirteen—hydrogen exchange mass spectrometry. In: Kelman Z (ed) Methods in enzymology. Academic Press, pp 335–356 10.1016/bs.mie.2015.06.035 PMC5858910 26791986

[CR40] Park JY, Qu C-X, Li R-R, Yang F, Yu X, Tian Z-M, Shen Y-M, Cai B-Y, Yun Y, Sun J-P et al (2019) Structural mechanism of the arrestin-3/JNK3 interaction. Structure 27:1162–1170.e1163 31080119 10.1016/j.str.2019.04.002

[CR41] Perry NA, Kaoud TS, Ortega OO, Kaya AI, Marcus DJ, Pleinis JM, Berndt S, Chen Q, Zhan X, Dalby KN et al (2019) Arrestin-3 scaffolding of the JNK3 cascade suggests a mechanism for signal amplification. Proc Natl Acad Sci USA 116:810–815 30591558 10.1073/pnas.1819230116 PMC6338856

[CR42] Perry-Hauser NA, Hopkins JB, Zhuo Y, Zheng C, Perez I, Schultz KM, Vishnivetskiy SA, Kaya AI, Sharma P, Dalby KN et al (2022) The two non-visual arrestins engage ERK2 differently. J Mol Biol 434:167465 35077767 10.1016/j.jmb.2022.167465 PMC8977243

[CR43] Qu C, Park JY, Yun MW, He Q-T, Yang F, Kim K, Ham D, Li R-R, Iverson TM, Gurevich VV et al (2021a) Scaffolding mechanism of arrestin-2 in the cRaf/MEK1/ERK signaling cascade. Proc Natl Acad Sci USA 118:e2026491118 34507982 10.1073/pnas.2026491118 PMC8449410

[CR44] Qu C, Park JY, Yun MW, He QT, Yang F, Kim K, Ham D, Li RR, Iverson TM, Gurevich VV et al (2021b) Scaffolding mechanism of arrestin-2 in the cRaf/MEK1/ERK signaling cascade. Proc Natl Acad Sci USA 118:e2026491118 10.1073/pnas.2026491118 PMC8449410 34507982

[CR45] Seyedabadi M, Gharghabi M, Gurevich EV, Gurevich VV (2021) Receptor-arrestin interactions: the GPCR perspective. Biomolecules 11:218 33557162 10.3390/biom11020218 PMC7913897

[CR46] Shukla AK, Manglik A, Kruse AC, Xiao K, Reis RI, Tseng W-C, Staus DP, Hilger D, Uysal S, Huang L-Y et al (2013a) Protein Data Bank. Structure of active beta-arrestin1 bound to V2Rpp (https://www.rcsb.org/structure/4JQI) [DATASET] 10.1038/nature12120 PMC3654799 23604254

[CR47] Shukla AK, Manglik A, Kruse AC, Xiao K, Reis RI, Tseng W-C, Staus DP, Hilger D, Uysal S, Huang L-Y et al (2013b) Structure of active β-arrestin-1 bound to a G-protein-coupled receptor phosphopeptide. Nature 497:137–141 23604254 10.1038/nature12120 PMC3654799

[CR48] Shukla AK, Westfield GH, Xiao K, Reis RI, Huang L-Y, Tripathi-Shukla P, Qian J, Li S, Blanc A, Oleskie AN et al (2014) Visualization of arrestin recruitment by a G-protein-coupled receptor. Nature 512:218–222 25043026 10.1038/nature13430 PMC4134437

[CR49] Smith JS, Pack TF, Inoue A, Lee C, Zheng K, Choi I, Eiger DS, Warman A, Xiong X, Ma Z et al (2021) Noncanonical scaffolding of G(alphai) and beta-arrestin by G protein-coupled receptors. Science 371:eaay1833 33479120 10.1126/science.aay1833 PMC8005335

[CR50] Smith JS, Rajagopal S (2016) The β-arrestins: multifunctional regulators of G protein-coupled receptors. J Biol Chem 291:8969–8977 26984408 10.1074/jbc.R115.713313 PMC4861465

[CR51] Srivastava A, Gupta B, Gupta C, Shukla AK (2015) Emerging functional divergence of β-arrestin isoforms in GPCR function. Trends Endocrinol Metab 26:628–642 26471844 10.1016/j.tem.2015.09.001

[CR52] Staus DP, Hu H, Robertson MJ, Kleinhenz ALW, Wingler LM, Capel WD, Latorraca NR, Lefkowitz RJ, Skiniotis G (2020) Structure of the M2 muscarinic receptor–β-arrestin complex in a lipid nanodisc. Nature 579:297–302 31945772 10.1038/s41586-020-1954-0 PMC7367492

[CR53] Wisler JW, Xiao K, Thomsen ARB, Lefkowitz RJ (2014) Recent developments in biased agonism. Curr Opin Cell Biol 27:18–24 24680426 10.1016/j.ceb.2013.10.008 PMC3971386

[CR54] Yang F, Yu X, Liu C, Qu C-X, Gong Z, Liu H-D, Li F-H, Wang H-M, He D-F, Yi F et al (2015) Phospho-selective mechanisms of arrestin conformations and functions revealed by unnatural amino acid incorporation and 19F-NMR. Nat Commun 6:8202 26347956 10.1038/ncomms9202 PMC4569848

[CR55] Yun Y, Kim DK, Seo M-D, Kim K-M, Chung KY (2015) Different conformational dynamics of β-arrestin1 and β-arrestin2 analyzed by hydrogen/deuterium exchange mass spectrometry. Biochem Biophys Res Commun 457:50–57 25542150 10.1016/j.bbrc.2014.12.079

[CR56] Zhai R, Wang Z, Chai Z, Niu X, Li C, Jin C, Hu Y (2023) Distinct activation mechanisms of beta-arrestin-1 revealed by (19)F NMR spectroscopy. Nat Commun 14:7865 38030602 10.1038/s41467-023-43694-1 PMC10686989

[CR57] Zhan X, Gimenez LE, Gurevich VV, Spiller BW (2011a) Protein Data Bank. Structure of bovine beta-arrestin 2. (https://www.rcsb.org/structure/3P2D) [DATASET]

[CR58] Zhan X, Gimenez LE, Gurevich VV, Spiller BW (2011b) Crystal structure of arrestin-3 reveals the basis of the difference in receptor binding between two non-visual subtypes. J Mol Biol 406:467–478 21215759 10.1016/j.jmb.2010.12.034 PMC3034793

[CR59] Zhao Y, Fan Y, Daolai Z, Zhixin L, Amy L, Chuan L, Peng X, Xiao Y, Jin-Peng S (2017) Phosphorylation of G protein-coupled receptors: from the barcode hypothesis to the flute model. Mol Pharmacol 92:201 28246190 10.1124/mol.116.107839

[CR60] Zhou XE, He Y, de Waal PW, Gao X, Kang Y, Van Eps N, Yin Y, Pal K, Goswami D, White TA et al (2017) Identification of phosphorylation codes for arrestin recruitment by G protein-coupled receptors. Cell 170:457–469.e413 28753425 10.1016/j.cell.2017.07.002 PMC5567868

