# Supplementary material for: Molecular mechanism of β-arrestin-2 pre-activation by phosphatidylinositol 4,5-bisphosphate
Source: EMBO Rep. 2024 Sep 6;25(10):12. doi: 10.1038/s44319-024-00239-x (PMC11467438; doi:10.1038/s44319-024-00239-x)
Supplement: Supplementary file 1 — Table EV1 [file 44319_2024_239_MOESM1_ESM.docx]

**Table EV1. Summary of the PIP_2_- or V2Rpp-binding effects on HDX-MS profiles of selected regions**

|  | PIP_2_-binding | | | | | V2Rpp-binding |
| --- | --- | --- | --- | --- | --- | --- |
|  | WT | 1-394 | Y64A | L280G | E315A | WT |
| 1-19: βl - βl/βll loop | I | I | I | X | X | D |
| 62-69: N-terminal half of finger loop | D | X | D | X | X | I |
| 70-76: C-terminal half of finger loop | D | D | D | D | D | D |
| 75-81: βVI | D | D | X | D | X | X |
| 168-186: βX - βXl | D | D | D | D | X | X |
| 195-201: N-terminal of 197-loop | D | D | D | D | X | D |
| 246-250: C-loop | D | D | D | D | D | X |
| 281-291: Lariat loop | D | D | D | N/D | X | D |
| 292-302: Gate loop | I | I | I | X | X | I |
| 303-306: The linker  between gate loop and back loop | D | D | D | D | X | D |
| 324-338: βXVlll - 344-loop | D | D | D | D | D | D |
| 382-389: βXX | I | I | I | X | X | I |

*I: increased HDX upon PIP_2_- or V2Rpp-binding
*D: Decreased HDX upon PIP_2_- or V2Rpp-binding
*X: No changes
*N/D: not detected due to the lack of identified peptides
