# Supplementary material for: Molecular mechanism of β-arrestin-2 pre-activation by phosphatidylinositol 4,5-bisphosphate
Source: EMBO Rep. 2024 Sep 6;25(10):12. doi: 10.1038/s44319-024-00239-x (PMC11467438; doi:10.1038/s44319-024-00239-x)
Supplement: Supplementary file 4 — Expanded View Figures [file 44319_2024_239_MOESM4_ESM.pdf]

Expanded View Figures

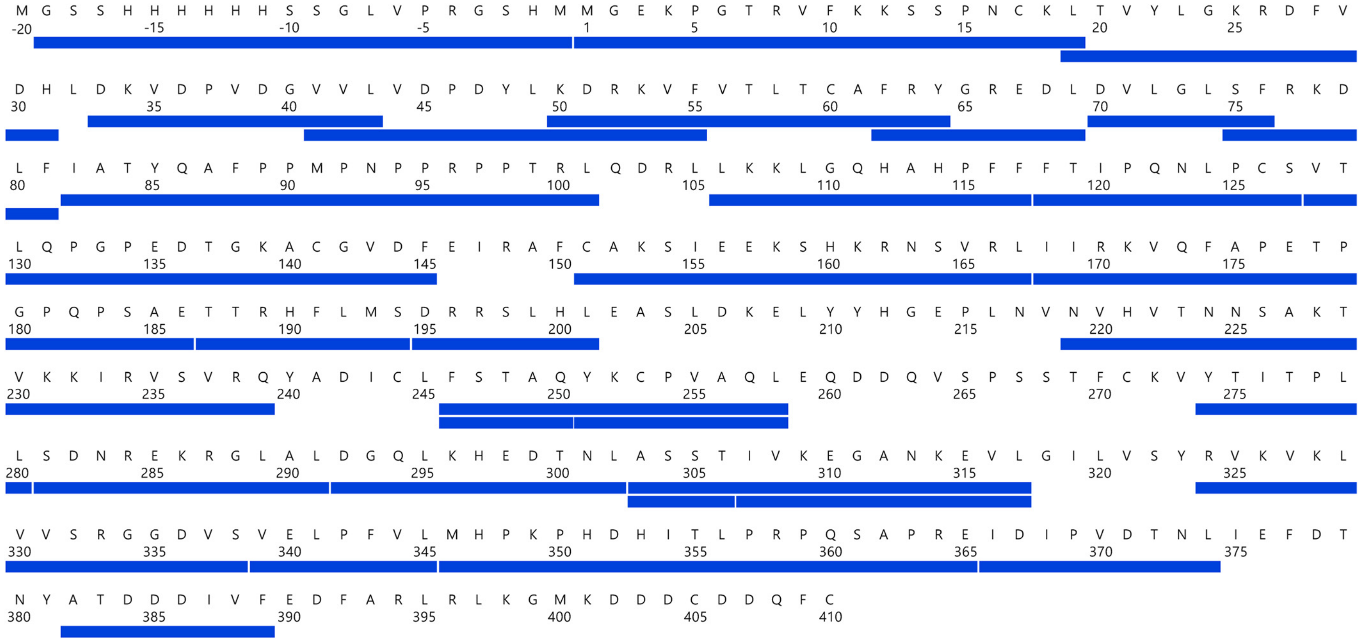

Figure EV1. Sequence coverage map of wild-type β-arrestin-2 (βarr2).

The blue bars indicate analyzed peptic peptides.

© The Author(s)

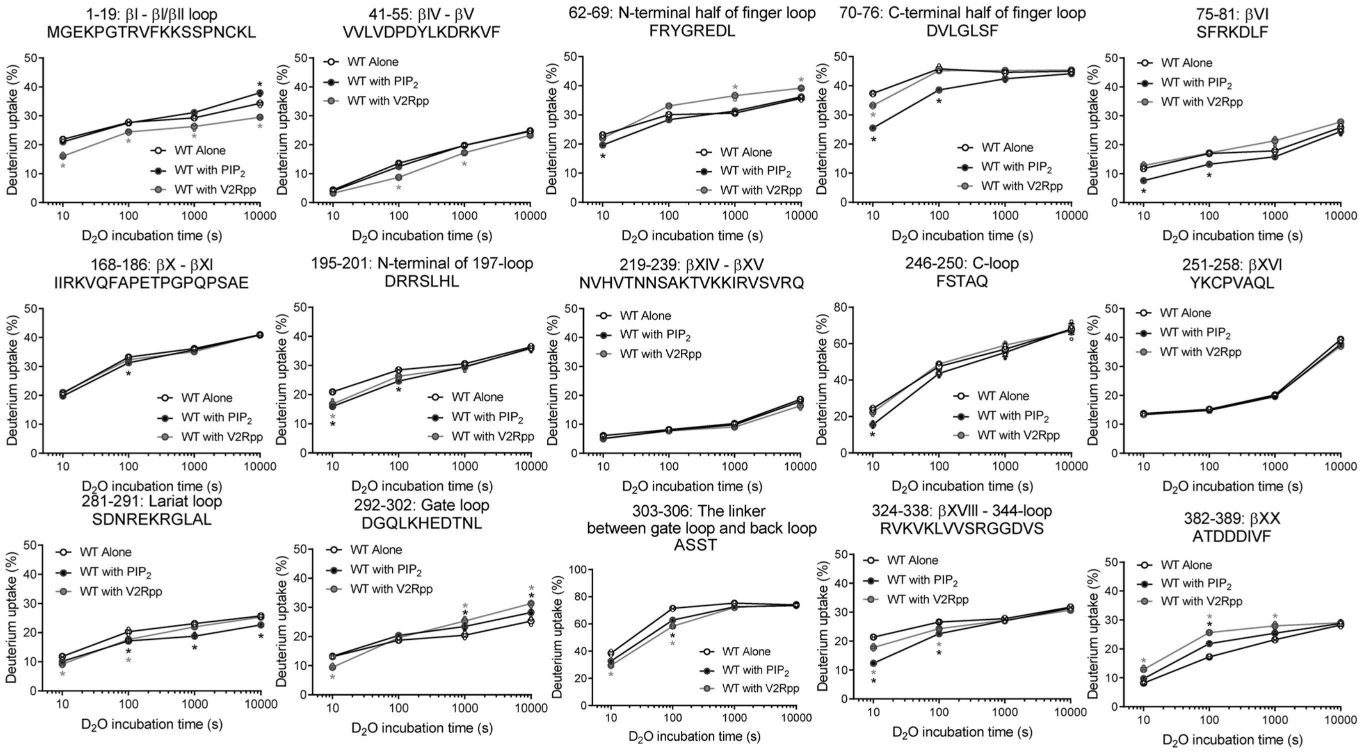

Figure EV2. Deuterium uptake plots of selective peptides of WT βarr2 with or without V2Rpp or PIP_2 co-incubation.

Results were derived from three independent experiments. The statistical significance of the differences was determined using Student's t test (*$P < 0.05$). Exact *p*-values are provided in Dataset EV1. Data are presented as mean ± standard error of the mean. Black or grey * indicates statistically significant difference between apo WT βarr2 and PIP_2-bound WT βarr2 or V2Rpp-bound WT βarr2, respectively. Smaller symbols indicate each data point.

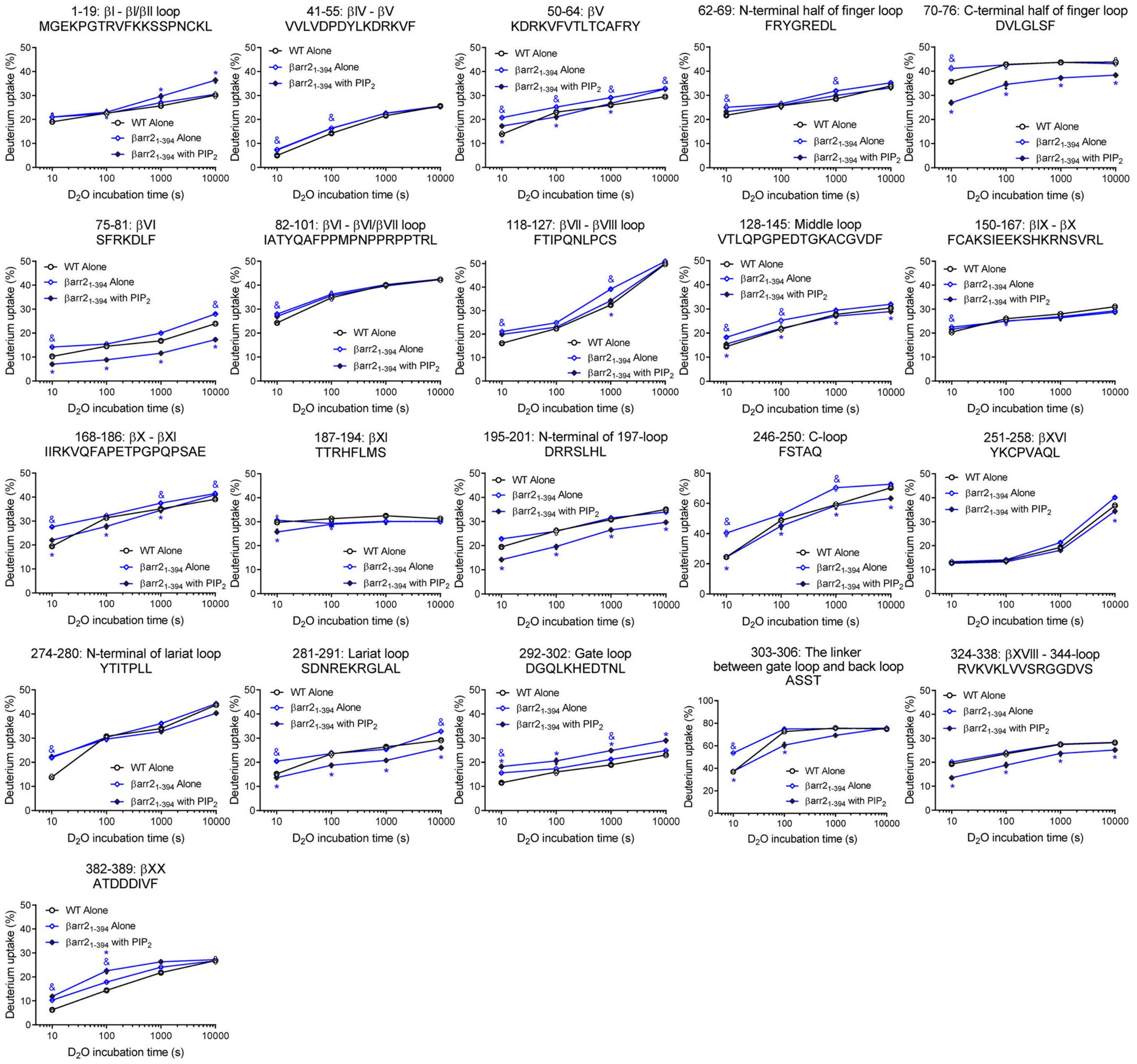

Figure EV3. Deuterium uptake plots of selective peptides of apo WT βarr2 and βarr2_1-394 with or without PIP_2 co-incubation.

Results were derived from three independent experiments. The statistical significance of the differences was determined using Student's *t* test ($^{\&,*}P < 0.05$). Exact *p*-values are provided in Dataset EV1. & indicates statistically significant difference between apo WT βarr2 and apo βarr2_1-394. * indicates statistically significant difference between apo and PIP_2-bound βarr2_1-394. Data are presented as mean ± standard error of the mean. Smaller symbols indicate each data point.

© The Author(s)

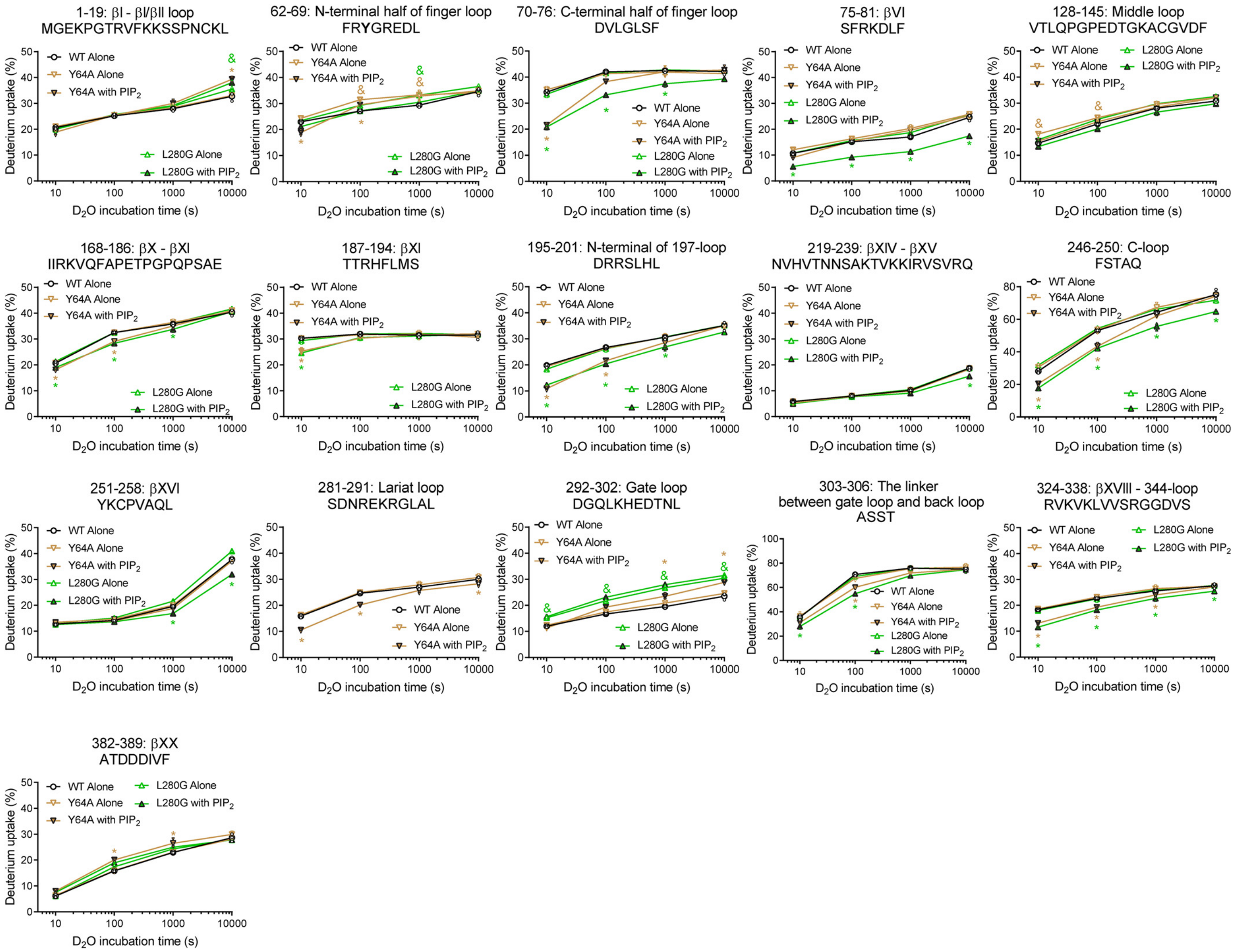

Figure EV4. Deuterium uptake plots of selective peptides of apo WT βarr2 and Y64A and L280G with or without PIP_2 co-incubation.

Results were derived from three independent experiments. The statistical significance of the differences was determined using Student's *t* test ($^{\&,*}P < 0.05$). Exact *p*-values are provided in Dataset EV1. Yellow green & indicates statistically significant difference between apo WT βarr2 and apo Y64A or L280G, respectively. Yellow or green * indicates statistically significant difference between apo and PIP_2-bound Y64A or L280G, respectively. Data are presented as mean ± standard error of the mean. Smaller symbols indicate each data point.

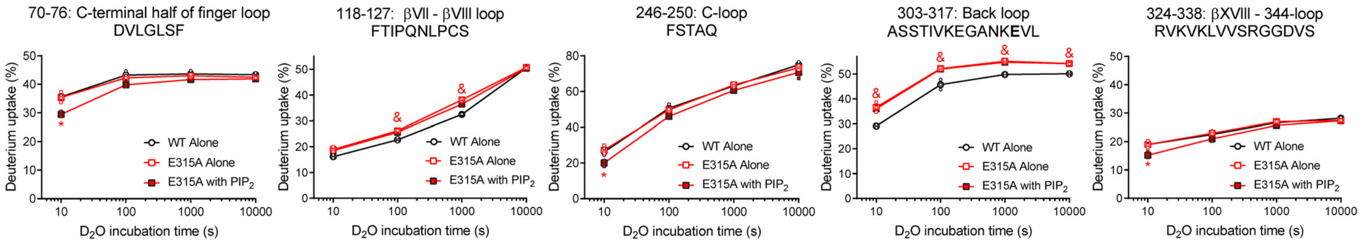

Figure EV5. Deuterium uptake plots of selective peptides of apo WT βarr2 and E315A with or without PIP_2 co-incubation.

Results were derived from three independent experiments. The statistical significance of the differences was determined using Student's *t* test ($^{\&,*}P < 0.05$). Exact *p*-values are provided in Dataset EV1. & indicates statistically significant difference between apo WT βarr2 and apo E315A. * indicates statistically significant difference between apo and PIP_2-bound E315A. Data are presented as mean ± standard error of the mean. Smaller symbols indicate each data point.

 © The Author(s)
